# Uniqueness of Heilongjiang Mandarin Fish (*Siniperca chuatsi*): Identifying Growth-Related Functional Loci Through Whole-Genome Analysis Across Four Geographic Populations

**DOI:** 10.3390/vetsci13010055

**Published:** 2026-01-07

**Authors:** Binglin Chen, Zhiying Zou, Wei Xiao, Hong Yang, Ying Zhang, Yongju Luo, Zhongbao Guo, Bo Li, Qingyun Wang, Kai Cui, Xiang Wang, Zhonggui Xie

**Affiliations:** 1 Freshwater Fisheries Research Center, Chinese Academy of Fishery Sciences, Wuxi 214081, China; chenbinglin@ffrc.cn (B.C.); zouzhiying@ffrc.cn (Z.Z.); xiaowei@ffrc.cn (W.X.); 2 Heilongjiang Fisheries Research Institute, Chinese Academy of Fishery Sciences, Harbin 150076, China; 940126179@qq.com; 3 Guangxi Zhuang Autonomous Region Fisheries Research Institute, Nanning 530021, China; lfylzc123@163.com (Y.L.); 65362393@qq.com (Z.G.); 4 Fisheries Research Institute, Wuhan Academy of Agricultural Sciences, Wuhan 430207, China; 352630431@qq.com (B.L.); 418438919@qq.com (Q.W.); 5 Fisheries Research Institute, Anhui Academy of Agricultural Sciences, Hefei 230022, China; cuikai66@163.com (K.C.); 18009697940@163.com (X.W.); 6 Hunan Fisheries Science Institute, Changsha 410153, China; 23692569@qq.com

**Keywords:** *Siniperca chuatsi*, population uniqueness, growth, functional loci

## Abstract

To analyze growth trait differences and genetic characteristics of *Siniperca chuatsi* from distinct geographic populations, whole-genome resequencing was performed on 90 samples from Heilongjiang (HLJ), Hubei (HB), Hunan (HN), and Anhui (AH), and for the first time, population-unique growth-related loci were detected. Population structure analysis indicated that the four populations were genetically distinct (K = 4). A preliminary Genome-Wide Association Study (GWAS) revealed 26 significant growth-related SNPs linked to 158 potential functional genes, which were primarily enriched in Metabolic pathways, the NOD-like receptor signaling pathway, and Necroptosis, suggesting their possible roles in growth regulation. Non-tag SNP capture within functional genes yielded 24 known significant loci and 118 potentially linked loci. On this basis, one-way ANOVA ultimately identified 13 SNPs significantly associated with growth, and their advantageous genotypes were characterized. Notably, this study revealed, for the first time, that the HLJ population possesses six population-unique genotypes significantly linked to superior growth traits, which may represent population-restricted candidate markers associated with growth-related variation. The results of this study provide new candidate molecular markers and supporting data that may inform future breeding strategies pending further validation.

## Simple Summary

This study aimed to analyze growth differences and genetic characteristics in Mandarin fish (*Siniperca chuatsi*) from different regions. We performed genome sequencing on 90 fish from four geographic populations. For the first time, we identified unique genetic markers linked to growth that were specific to these populations. Our analysis confirmed that the four populations are genetically distinct. We initially discovered 26 key genetic variations significantly associated with growth, potentially influencing it through metabolic pathways and other biological processes. Further analysis pinpointed 13 significant genetic markers and identified their beneficial types associated with superior growth. Notably, the Heilongjiang population uniquely possessed six of these beneficial genetic types, which may represent population-restricted candidate markers relevant to growth-related breeding potential. These findings provide new candidate molecular markers and supporting data that may inform future breeding strategies and require further validation under controlled conditions.

## 1. Introduction

Molecular markers primarily refer to polymorphic bases or fragments within DNA sequences. First-generation molecular markers, such as microsatellites (SSRs) and restriction fragment length polymorphisms (RFLPs), advanced genetic analysis to the nucleic acid level [1,2]. With the advancement of high-throughput technologies, single-nucleotide polymorphisms (SNPs) have gradually become the predominant type of molecular marker in molecular marker research. Compared with other types of molecular markers, SNPs require larger sample sizes and provide higher accuracy, and have been effectively applied in various biological fields, including growth [3], disease resistance [4], and environmental adaptability [5]. By analyzing significant differences in target traits among genotypes, genotypes significantly associated with superior traits can be identified and subsequently applied to studies on the mechanisms by which loci regulate traits or to selective breeding [6]. Currently, next-generation sequencing (NGS) technology has enabled the development and application of genome-wide SNP markers, which have made significant contributions to selective breeding and the improvement in economic traits in various aquaculture species, including Atlantic salmon (*Salmo salar*) [7], rainbow trout (*Oncorhynchus mykiss*) [8], and largemouth bass (*Micropterus salmoides*) [9].

Mandarin fish (*Siniperca chuatsi*) belongs to the order Perciformes, subfamily Sinipercinae, and genus *Siniperca*. With a long history of aquaculture, it is one of the major native species in China’s aquaculture industry and represents the most economically valuable species among the 11 recognized species of the genus *Siniperca*. Mandarin fish is distributed across multiple river basins in China, ranging from the Heilongjiang River Basin in the north to the Pearl River Basin in the south. Its primary habitats are concentrated in river basins south of the Yangtze River, particularly in regions such as Hubei, Hunan, Anhui, and Guangdong. In 2024, aquaculture production of Mandarin fish in China reached 470,000 tonnes, representing a 18% year-on-year increase, and the industry is experiencing rapid growth [10,11]. However, reports have indicated that with the expansion of farming scale, a decline in germplasm genetic diversity has been observed under successive multi-generation selection [12], and significant genetic differentiation between cultured and wild populations has emerged [13]. The expansion of aquaculture scale inevitably increases the frequency of contact among populations from different geographic regions, thereby raising the likelihood of germplasm admixture and excessive morphological variation between populations, which together pose dual challenges to germplasm conservation and the sustainable development of the aquaculture industry.

Growth is a critical trait in aquaculture species, influenced by a variety of factors including strain, farming environment, and nutritional composition. Among these, strain occupies a primary position in the aquaculture production chain. Superior strains can enhance adaptability to adverse conditions and contribute to economically important traits by either promoting positive effects or mitigating negative effects [14]. Mandarin fish exhibits a relatively large size and rapid growth; however, significant within-population variation in growth rate is observed during aquaculture, similar to other economically important perciform fishes, such as Nile tilapia (*Oreochromis niloticus*) [15]. Previous studies have made notable progress in understanding the genetic basis of growth-related traits in mandarin fish. Candidate gene approaches were employed to investigate growth-associated genes, such as growth hormone and other metabolism-related genes, and significant associations between gene polymorphisms and growth traits were reported [16]. With the development of high-throughput sequencing technologies, genomic resources for Mandarin fish have been substantially improved, including the availability of a chromosome-level reference genome [17]. Based on these resources, several studies have constructed high-density linkage maps and identified growth-related loci, providing valuable insights into the genomic regions potentially underlying growth variation [18]. In addition, transcriptomic and microRNA-based analyses have revealed regulatory genes and pathways involved in muscle development and growth regulation in Mandarin fish [19]. More recently, genome-wide association studies and whole-genome resequencing analyses have been applied to Mandarin fish, leading to the identification of growth-related SNPs, candidate genes, and signatures of selection among different populations [20]. However, despite these advances, most existing studies have been conducted within single populations or limited breeding stocks, and comprehensive genome-wide comparisons of growth-associated loci across geographically distinct populations remain scarce. In particular, the extent to which population-specific SNPs contribute to growth variation and their potential implications for selective breeding and germplasm conservation have not been fully explored. By comparison, a genome-wide analysis of Pacific white shrimp (*Litopenaeus vannamei*) identified population-specific SNPs and selection signatures among geographically distinct breeding populations, and these loci were suggested to reflect population-level adaptation and artificial selection during domestication and breeding programs [21]. Such population-specific variants have been proposed as informative markers for distinguishing breeding stocks, evaluating genetic resources, and guiding breeding strategies. Furthermore, due to the wide distribution of Mandarin fish across diverse habitats, regional differences have increased the difficulty of accurately assessing its growth performance [22]. Therefore, the evaluation and rational utilization of growth traits in populations among geographic populations remain key areas in Mandarin fish research.

In this study, whole-genome resequencing was performed on four Mandarin fish populations from Heilongjiang, Hubei, Hunan, and Anhui to investigate the genetic characteristics and diversity differences among geographic populations. To achieve more comprehensive and accurate GWAS results, significant GWAS loci were analyzed to explore potentially linked loci within functional genes, ultimately leading to the identification of growth-related candidate molecular markers and their superior genotypes. Based on these analyses, this study conducted the first identification of population-unique SNPs across different Mandarin fish populations and subsequently assessed the selective breeding potential of each geographic population. This study provides foundational data for germplasm evaluation and quality improvement in Mandarin fish with a focus on growth traits.

## 2. Materials and Methods

### 2.1. Experimental Animals and Sample Collection

This study was approved by the Animal Welfare and Ethics Committee of the Freshwater Fisheries Research Center, Chinese Academy of Fishery Sciences (Approval No. SYXK [SU] 2017-0007). In 2024, samples were collected from four geographic populations: Heilongjiang (HLJ), Anhui (AH), Hunan (HN), and Hubei (HB), with sample sizes of 20, 20, 20, and 30, respectively. All individuals sampled were produced in the year 2024. They were collected from conservation breeding facilities and maintained under standardized conditions as described by Xiao et al. [23].

The HLJ population (17.4 ± 3.5 g, sampled June 2024) originated from the Heilongjiang River (Heilongjiang River basin), Heilongjiang Province, China, and is conserved by the Heilongjiang Fisheries Research Institute, Chinese Academy of Fishery Sciences. The AH population (18.7 ± 4.1 g, sampled July 2024) was sourced from Caizi Lake (Zongyang Changhe River basin, a tributary of the Yangtze River), Anhui Province, China, and is conserved by the Fisheries Research Institute, Anhui Academy of Agricultural Sciences. The HN population (30.6 ± 5.5 g, sampled July 2024) originated from Dongting Lake (part of the Yangtze River basin), Hunan Province, China, and is maintained by the Hunan Fisheries Science Institute. The HB population (625.2 ± 44.3 g, sampled October 2024) was sourced from the Wuhan section of the Yangtze River basin, Hubei Province, China, and is conserved by the Fisheries Research Institute, Wuhan Academy of Agricultural Sciences. During sampling, selected individuals were anesthetized in water at 25 °C using MS-222 at a concentration of 13.5 g/m^3^ to minimize stress-related effects on the experiment. Body weight was measured, and caudal fin tissue was collected and preserved at −80 °C for subsequent genomic DNA extraction, library construction, and whole-genome sequencing analysis.

### 2.2. Whole-Genome DNA Extraction, Library Construction and Sequencing

Whole-genome DNA was extracted using the TIANamp Marine Animals DNA Kit (TIANGEN Biotech, Beijing, China). DNA integrity was assessed by 1% agarose gel electrophoresis, and DNA purity and concentration were quantified using a Qubit 4.0 fluorometer (Invitrogen, Carlsbad, CA, USA). Genomic DNA (gDNA) from each sample was used for library preparation following the protocol of the YZSeq Tn5 Library Prep Kit (ShadowGene, Wuhan, China). In a 96-well plate, gDNA was digested using EcoRI and HaeIII restriction enzymes. Adapters A1 and A2 (25 pmol/well) were then ligated to the digested DNA. DNA purification was performed using 1% agarose gel electrophoresis followed by PCR purification with a kit from NEB (Ipswich, MA, USA). The purified products were then amplified by PCR, and sequencing was carried out on the Illumina NovaSeq 6000 platform (Illumina, San Diego, CA, USA) using the PE150 strategy.

### 2.3. Sequencing Data Processing and Polymorphism Analysis

Raw sequencing data were quality-controlled and filtered using fastp (version 0.23.2), including the removal of adapter sequences, low-quality bases, and reads with a high proportion of unidentified nucleotides [24]. The reference genome and annotation files of Mandarin fish (ASM2008510v1) were obtained from National Center for Biotechnology Information. The reference genome index was built using BWA (version 0.7.17) and clean reads were aligned to the reference genome for sequence mapping and polymorphism detection. SNP loci were extracted using Picard (version 2.20.7) and GATK (version 4.1.8.1) [25,26]. Based on the extracted SNPs, further stringent filtering of polymorphism results was conducted using VCFtools (version 0.1.17), and SNPs were retained if they met the following criteria: minor allele frequency (MAF) > 0.1, sample missing rate < 10%, and Hardy–Weinberg equilibrium *p*-value > 1 × 10^−6^ [27].

### 2.4. Genetic Distance, Genetic Diversity and Population Structure Analysis

Based on the high-quality SNPs retained from the above procedures, pairwise genetic distances between samples were calculated using PLINK (version 1.9). The resulting genetic distance matrix was analyzed and a neighbor-joining (NJ) tree was constructed using MEGA (version 11.0.0). To evaluate the genetic diversity of different populations, observed heterozygosity (Ho), expected heterozygosity (He), nucleotide diversity (Pi), and polymorphic information content (PIC) for each SNP locus were calculated using PLINK (version 1.9). Population structure was inferred by ADMIXTURE (version 1.3.0), where cross-validation error (CV error) and maximum likelihood values for different numbers of clusters (K) were used to assist in determining the optimal K for the studied populations.

### 2.5. Genome-Wide Association Analysis of Body Weight Traits

Following the method of [28,29], phenotypic correction across populations was conducted using the single-step GBLUP (ssGBLUP) method to minimize the influence of inter-population growth cycle differences. To further reduce potential confounding effects arising from differences in sampling time and growth stage, phenotypic adjustment was performed prior to genome-wide association analysis. The model was defined as follows:

y=Xb+Za+e

where *y* denotes the vector of individual phenotypic observations; *b* represents fixed effects, with *X* being the incidence matrix relating them to *y* (including population, age, location, and season); *a* denotes the vector of additive genetic effects (breeding values), with *Z* as its incidence matrix; and *e* is the vector of residuals. The residuals obtained from this model, representing individual-level body weight variation after removing systematic environmental and population-level effects, were used as input phenotypes for all subsequent GWAS analyses.

Body weight and genotype data from the four Mandarin fish populations were analyzed using the GAPIT (version 3.0) package in R [30]. Three statistical models were employed, including the Mixed Linear Model (MLM), Fixed and Random Model Circulating Probability Unification (FarmCPU), and Bayesian-information and Linkage-disequilibrium Iteratively Nested Keyway (BLINK). In all models, genetic relatedness among individuals was accounted for through the genomic kinship matrix. To control for multiple testing, Bonferroni correction was applied, and SNPs significantly associated with body weight were identified.

### 2.6. Gene Annotation and Functional Enrichment Analyses

Based on the annotated genome of *Siniperca chuatsi* (ASM2008510v1) from NCBI, gene annotation of each growth-related SNP locus was performed using the R package GenomicRanges (version 1.58.0) [31]. Subsequently, genes harboring all growth-related SNP loci as well as population-specific loci were subjected to Gene Ontology (GO) annotation and Kyoto Encyclopedia of Genes and Genomes (KEGG) enrichment analyses using DIAMOND (version 2.0.15). Enriched GO terms and KEGG pathways with *p* < 0.05 were considered significant.

### 2.7. Identification of Potentially Linked SNPs of Significant Loci, Screening of Growth-Related SNPs, and Identification of Population-Specific Loci

SNPs analyzed in the GWAS were derived via segmental genome-wide linkage disequilibrium (LD) analysis, and representative tag SNPs from each LD group were retained for subsequent analysis. Therefore, SNPs in high LD with these tag SNPs may have been excluded if they were not selected as representative loci. To further identify these SNPs, all SNPs within the annotated genes were subjected to LD analysis relative to significant SNPs from the GWAS results using R (stringr version 1.5.1; tidyr version 1.3.1). SNPs exhibiting r^2^ > 0.5 were regarded as potentially linked loci. These loci, together with the originally identified significant SNPs, were subsequently employed for analysis of growth-related superior genotypes.

One-way ANOVA followed by LSD tests was conducted to evaluate the association between potentially linked SNPs and body weight across four Mandarin fish populations (N = 90) using R packages data.table (version 1.17.0) and dplyr (version 1.14). Following the method of Chen et al. [3], a SNP was considered growth-related when body weight differed significantly among genotypes (*p* < 0.05). Genotype frequencies for each SNP in each population were determined using dplyr (version 1.14). SNPs were considered population-specific growth-related loci if their superior genotypes were present exclusively in one population. The body weight distribution of different genotypes at significant growth-related SNPs was subsequently visualized using ggplot2 (version 3.5.1) in R.

## 3. Results

### 3.1. Sequencing Results and Distribution of Polymorphic Loci

A total of 6,462,921,738 clean reads were obtained from 90 samples representing four populations (HLJ, AH, HN, and HB). The average mapping rate to the reference genome was 99.24%, with a mean GC content of 40.42%. The average Q20 and Q30 values were 99.43% and 98.03%, respectively, indicating high sequencing quality. Detailed sequencing statistics are provided in Supplementary Table S1. The sequencing data have been deposited in the SRA database under accession number PRJNA1252799.

**Figure 1:**
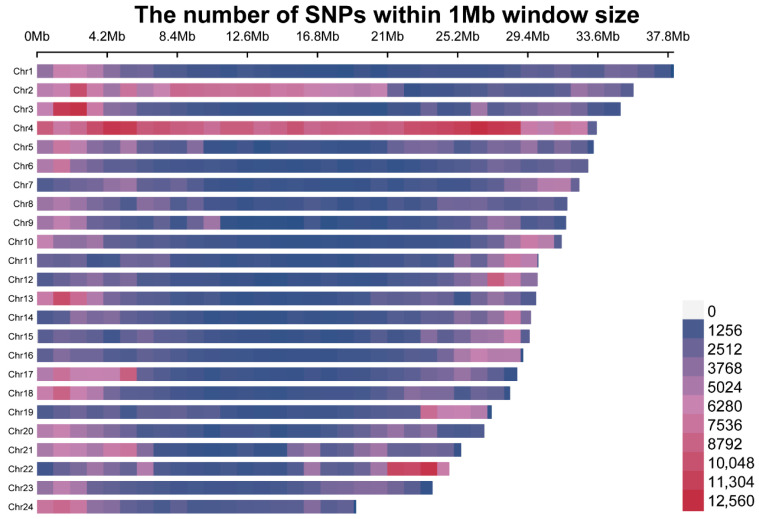
Chromosomal distribution of the final filtered SNP loci. Different colors represent the number of SNP loci.

A total of 17,059,782 SNP loci were initially identified. After preliminary filtering, 15,097,690 loci were retained. A final set of 1,939,223 high-quality SNP loci was obtained after additional filtering based on a minor allele frequency (MAF) > 0.1, missing rate < 10%, and Hardy–Weinberg equilibrium *p*-value > 1 × 10^−6^. Among these, transitions accounted for 58.00%, including 562,792 A/G loci (29.02%) and 561,959 C/T loci (28.98%). Transversions made up the remaining 42.00%, consisting of 207,819 A/C loci (10.72%), 252,865 A/T loci (13.04%), 146,809 C/G loci (7.57%), and 206,979 G/T loci (10.67%). The chromosomal distribution of these SNPs is shown in Figure 1. The number of loci per chromosome (chr) ranged from 52,541 to 310,724, differing by nearly sixfold. The highest and lowest SNP densities were observed on chr4 and chr23, respectively, while chr2 (175,030 loci) and chr22 (87,666 loci) showed the second highest and lowest counts.

### 3.2. Genetic Distance, Genetic Diversity, and Population Structure

**Figure 2:**
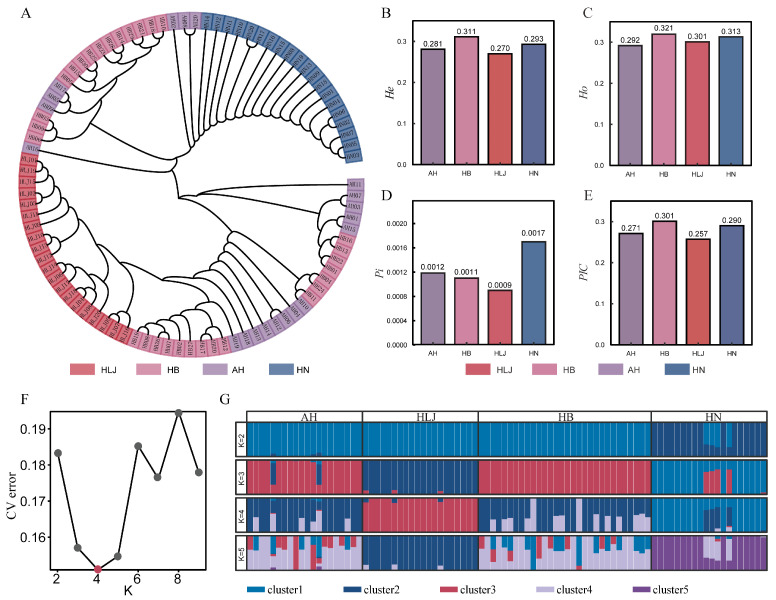
Genetic distance, genetic diversity, and population structure of the four populations. (**A**): Neighbor-joining tree of all samples. (**B**): Expected heterozygosity (He) in different populations. (**C**): Observed heterozygosity (Ho) in different populations. (**D**): Nucleotide diversity (Pi) in different populations. (**E**): Polymorphism information content (PIC) in different populations. (**F**): Cross-validation error values for different K values. The optimal value is indicated in red. (**G**): Population structure of different populations at different K values; different colors represent different clusters.

Based on Nei’s genetic distance, clustering analysis of the four populations (Figure 2A) showed that all 90 samples were grouped within a single dendrogram. Individuals from the HB and AH populations were distributed mainly in the upper and middle sections of the dendrogram, forming two overlapping regional clustering patterns. The HLJ population was positioned in the central section of the dendrogram, bridging the clustering patterns formed by HB and AH individuals. The HN population was clearly distinguished and primarily distributed in the lower section of the dendrogram. The genetic distances relative to the HLJ population were ranked as HB < AH < HN; for the AH population, as HB < HLJ < HN; for the HB population, as HLJ < AH < HN; and for the HN population, as HB < AH < HLJ. Genetic diversity results showed that the highest values of He, Ho, and PIC were all found in the HB population, while Pi was highest in the HN population. PIC values indicated that all four populations exhibited moderate polymorphism (Figure 2B–E). The results of population structure analysis indicated that the cross-validation error reached its minimum when K = 4, suggesting that the 90 individuals could be divided into four distinct genetic clusters. Specifically, the AH and HB populations were predominantly composed of Cluster 2 and Cluster 4; the HLJ population was mainly associated with Cluster 3; and the HN population was largely composed of Cluster 1, with a minor contribution from Cluster 2. These results were consistent with the clustering patterns observed in the neighbor-joining tree constructed for the four populations (Figure 2F,G).

### 3.3. GWAS of Growth Traits and Gene Annotation of Significant Loci

**Figure 3:**
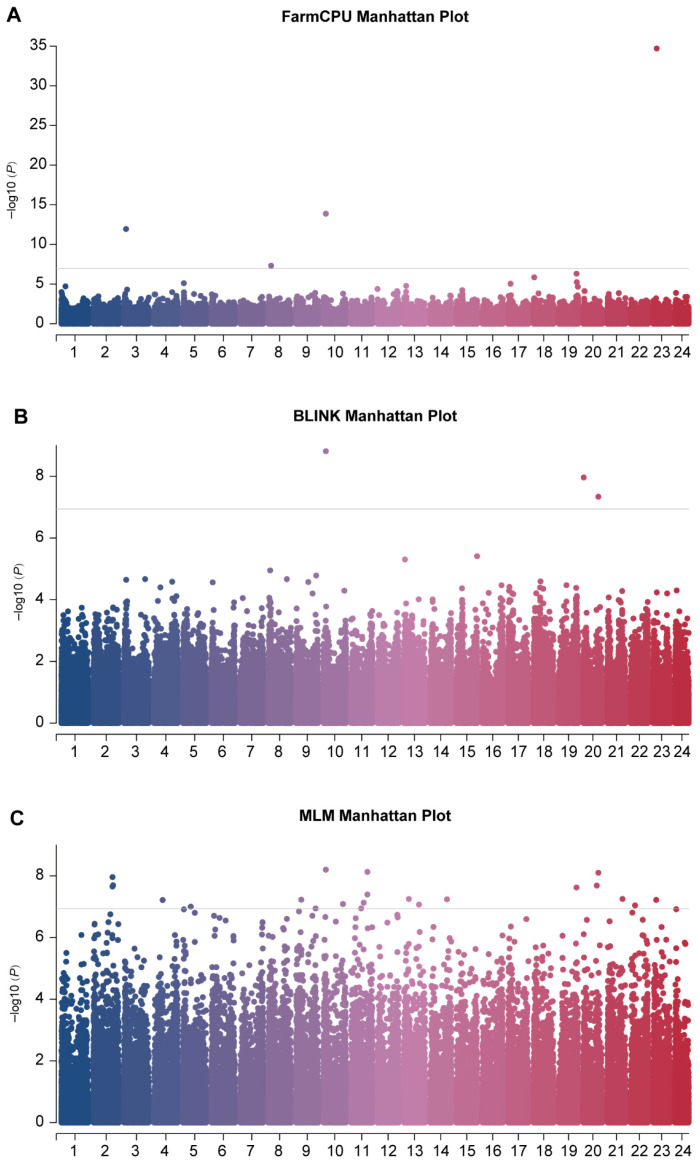
Manhattan plots of GWAS analyses based on three models: (**A**) FarmCPU, (**B**) BLINK, and (**C**) MLM. The horizontal line denotes the Bonferroni correction threshold.

Using FarmCPU, BLINK, and MLMs with Bonferroni correction, GWAS was performed for body weight traits. The three models identified 4, 3, and 22 significant loci, respectively (Figure 3). After eliminating overlapping loci, 26 SNPs significantly associated with body weight were retained. These loci were distributed across 16 chromosomes, including Chr2 (3), Chr3 (1), Chr4 (1), Chr5 (1), Chr8 (1), Chr9 (2), Chr10 (2), Chr11 (4), Chr13 (2), Chr14 (1), Chr19 (1), Chr20 (3), Chr21 (1), Chr22 (1), and Chr23 (2) (Table 1). Functional annotation within a ±50 kb window around each SNP revealed 158 potential functional genes (Supplementary Table S2).

### 3.4. GO and KEGG Annotation and Enrichment Analysis of Potential Functional Genes

**Figure 4:**
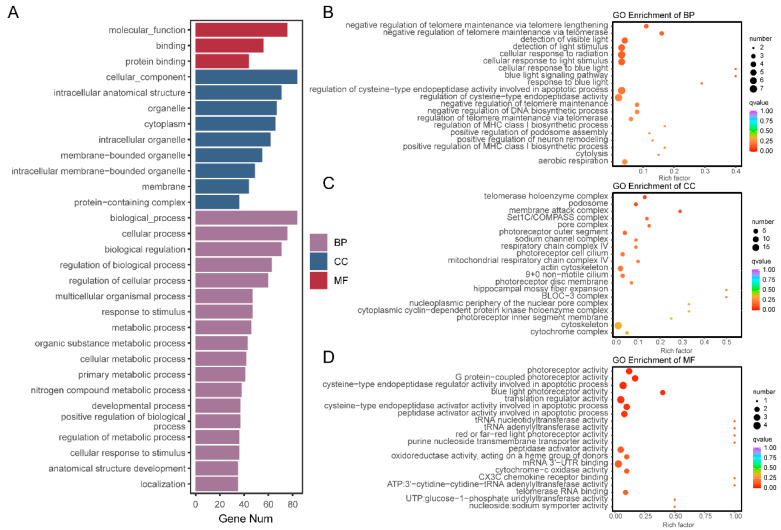
GO annotation and enrichment analysis of potential functional genes. (**A**) GO annotation. (**B**) Biological process (BP) enrichment. (**C**) Cellular component (CC) enrichment. (**D**) Molecular function (MF) enrichment.

GO annotation and enrichment analysis were performed for the potential functional genes. The annotation results mainly included molecular function (MF), cellular component (CC), and biological process (BP). Genes were primarily annotated to MF terms such as molecular function, binding, and protein binding; CC terms such as cellular component, intracellular anatomical structure, and organelle; and BP terms such as biological process, cellular process, and biological regulation (Figure 4A). For enrichment analysis, the top 20 most significant pathways were selected for BP, CC, and MF. The most significant BP terms were negative regulation of telomere maintenance via telomere lengthening, negative regulation of telomere maintenance via telomerase, detection of visible light, detection of light stimulus, and cellular response to radiation (Figure 4B). The top CC terms included telomerase holoenzyme complex, podosome, membrane attack complex, Set1C/COMPASS complex, and pore complex (Figure 4C). For MF, the most enriched terms were photoreceptor activity, G protein-coupled photoreceptor activity, cysteine-type endopeptidase regulator activity involved in apoptotic process, blue light photoreceptor activity, and translation regulator activity (Figure 4D).

**Figure 5:**
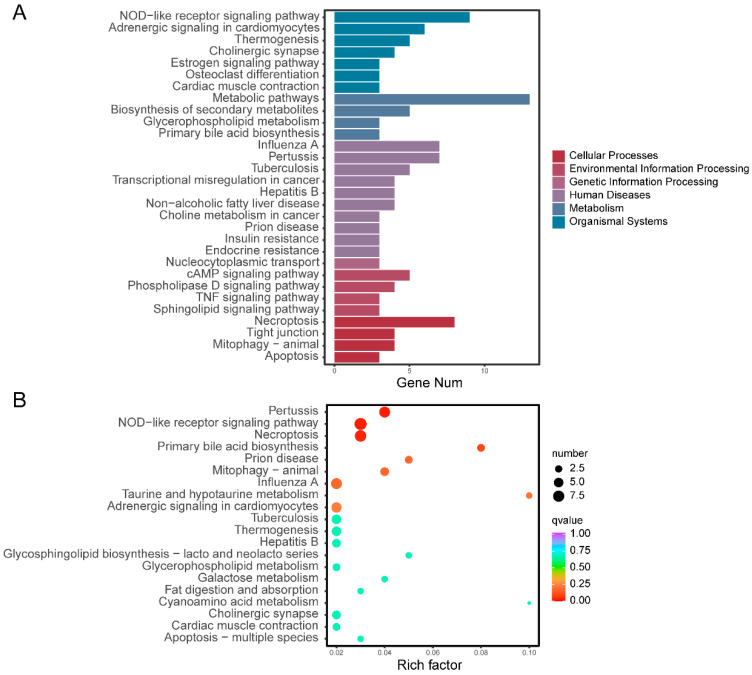
KEGG annotation and enrichment analysis of potential functional genes. (**A**) KEGG pathway annotation of genes. (**B**) KEGG enrichment analysis of genes.

KEGG annotation and enrichment analyses indicated that the potential functional genes were primarily concentrated in metabolic pathways, the NOD-like receptor signaling pathway, and necroptosis (Figure 5A). These genes were further found to be significantly enriched in pertussis, the NOD-like receptor signaling pathway, necroptosis, primary bile acid biosynthesis, prion disease, mitophagy, influenza A, taurine and hypotaurine metabolism, and adrenergic signaling in cardiomyocytes (Figure 5B).

### 3.5. Identification of Potentially Linked SNP Loci

A total of 17,764 SNP loci were detected within the 158 potential functional genes (Supplementary Table S3). By performing pairwise linkage disequilibrium analysis between these loci and the corresponding tag SNPs (i.e., the 26 significant SNPs identified in Section 3.3), loci with r^2^ > 0.5 were defined as potentially linked to the significant SNPs. Ultimately, 142 potentially linked SNP loci were obtained from all SNP loci within the potential functional genes (Supplementary Table S4), including 24 SNP loci already identified in GWAS as significantly associated with body weight (excluding two loci without gene annotation) and 118 potentially linked loci, which may be associated with body weight traits.

### 3.6. Association Analysis of Potentially Linked SNP Loci with Body Weight and Identification of Population-Unique Growth-Related SNPs

**Figure 6:**
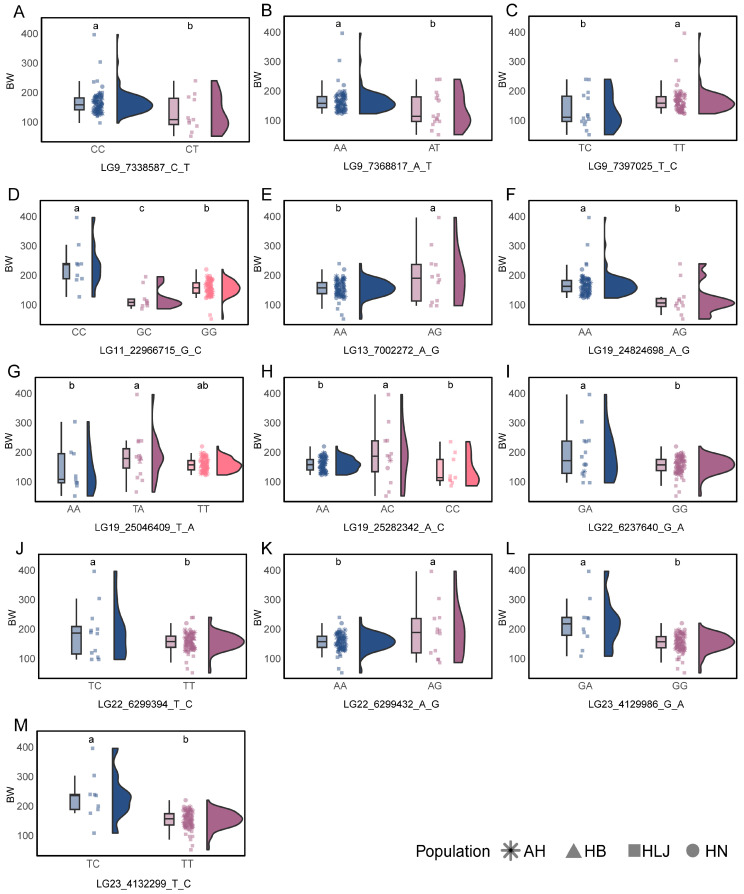
Association analysis of potentially linked SNP loci with body weight (*p* < 0.05). The shape of the peaks represents the density distribution of the samples, with different colors indicating genotypes of each SNP locus. The shape of the points represents different populations.

Using ANOVA and LSD tests, 13 of the 142 potentially linked SNP loci were found to be significantly associated with body weight, with superior genotypes: CC at LG9_7338587_C_T, AA at LG9_7368817_A_T, TT at LG9_7397025_T_C, CC at LG11_22966715_G_C, AG at LG13_7002272_A_G, AA at LG19_24824698_A_G, TA at LG19_25046409_T_A, AC at LG19_25282342_A_C, GA at LG22_6237640_G_A, TC at LG22_6299394_T_C, AG at LG22_6299432_A_G, GA at LG23_4129986_G_A, and TC at LG23_4132299_T_C (Figure 6).

Based on the population composition of the superior genotypes at each locus, six population-unique growth-related SNPs were identified, respectively, LG11_22966715_G_C, LG13_7002272_A_G, LG22_6299394_T_C, LG22_6299432_A_G, LG23_4129986_G_A, and LG23_4132299_T_C, all of which had superior genotypes exclusively derived from the HLJ population (Figure 6).

## 4. Discussion

Currently, genome-wide association studies (GWAS) have been widely reported in livestock and poultry such as domestic pigs (*Sus scrofa domestica*) [32], domestic chicken (*Gallus gallus domesticus*) [33], and cattle (*Bos taurus*) [34], as well as in various aquatic animals [35,36,37]. The process is efficient and accurate, but some limitations remain. First, it has been reported that the excessive conservativeness of the Bonferroni correction substantially increases the probability of false negatives in the results [38,39]. Second, the application of the Bonferroni correction requires the assumption that all tests are independent; however, in animal genome analyses, linkage disequilibrium exists among SNP loci, indicating that they are not entirely independent [40]. To satisfy the requirement of SNP independence, the genome is conventionally partitioned into contiguous segments of 50–100 Kb. Within each segment, linkage disequilibrium among SNP loci is analyzed, and tag SNPs are retained. These tag SNPs are subsequently used for model construction and Bonferroni correction. However, the retention of tag SNPs is random, and their positions are not entirely located within the putative functional regions of genes, such as promoters and coding sequence (CDS) [41]. For example, linkage disequilibrium exists between a hypothetical SNP (hereafter referred to as SNP_1_) located within the CDS region and another linked SNP (hereafter referred to as SNP_2_) situated in non-coding or other low-functional regions. However, SNP_2_ is retained as a tag SNP and subsequently excluded from downstream model analyses due to its lower potential functionality, resulting in the potential regulatory effect of SNP_1_ on the target phenotype being concealed. As a consequence, false negatives may be exhibited by linkage disequilibrium modules, a phenomenon documented repeatedly in the medical field [42,43]. In addition, SNPs obtained from GWAS analyses provide only positional information and do not indicate the optimal genotypes, which may constrain their application as molecular markers. To overcome this limitation, we combined potentially linked SNP identification with one-way ANOVA across different genotype groups, resulting in 24 SNPs previously confirmed to be significantly associated with body weight and 118 potentially linked loci. From these, 13 SNPs significantly associated with body weight were identified and their superior genotypes determined, substantially improving the accuracy of marker coverage and their utility in molecular breeding.

At present, recent research on Mandarin fish has primarily focused on dietary optimization [44,45], sex determination [46,47], and disease resistance mechanisms [48,49]. Studies on different geographical populations have primarily focused on genetic diversity and population structure [22]. However, no studies have been reported on the evaluation of growth-related breeding potential in different populations. In this study, the genetic structure of Mandarin fish from four different populations was assessed, growth-related functional SNPs were rigorously identified, and population- unique analyses of these loci were conducted. The HLJ population, identified as a relatively rare group within Mandarin fish, was shown to exhibit independence in population structure in this study, which is consistent with the population structure pattern reported by of Cui et al. [22]. In the subsequent identification of population-unique growth-related loci, six loci were discovered, and all of these loci were found exclusively in the HLJ population, which may be related to the HLJ population’s isolated aquatic habitat. The Heilongjiang basin, situated at the Sino-Russian border in Heilongjiang Province, is geographically isolated from the Yangtze River basin, which represents the principal habitat of Mandarin fish. Consequently, genotypes exclusive to the HLJ population and lacking in other populations may contribute to variations in phenotypic traits, and this supposition is supported by the results of the present study. Within the six population-unique loci described above, genotypes linked to superior growth performance have been detected exclusively in the HLJ population, but it should be noted that body weight differed substantially among populations due to differences in sampling time and growth stage. Although phenotypic correction and mixed-model-based GWAS were applied to control for population structure, the population-specific nature of these genotypes should be interpreted cautiously. The identification of HLJ population-unique superior genotypes indicates population-restricted candidate markers associated with growth-related variation, rather than definitive causal variants for selection. Further validation under common-garden conditions or controlled breeding experiments will be required to disentangle genetic effects from potential genotype–environment interactions. At present, selective breeding of Mandarin fish has been predominantly conducted through intraspecific hybridization within the HB, AH, and HN populations, or interspecific hybridization between Mandarin fish and Spotted mandarin fish (*Siniperca scherzeri*) [50], with no reports indicating participation of the HLJ population in such breeding programs. Considering its unique geographical location and exclusive genotypes associated with superior growth performance, the HLJ population is regarded as having great potential for selective breeding. However, necessary conservation assessments should be conducted prior to introduction and utilization, with strict measures implemented to prevent farm escapees and interpopulation admixture. Moreover, it has been shown that organisms inhabiting low-temperature environments can enhance their cold tolerance by regulating mitochondrial function [51]. The HLJ population has historically inhabited the Heilongjiang basin, which spans the cold-temperate and temperate zones and is characterized by low water temperatures and considerable seasonal fluctuations. Therefore, the HLJ population may show increased adaptability to environmental variation. Environmental temperature is one of the most important ecological factors influencing fish physiology and growth, primarily through the regulation of the growth hormone–insulin-like growth factor (GH–IGF) endocrine axis. Extensive evidence across teleost species has shown that seasonal and experimental changes in water temperature are closely associated with variation in somatic growth, circulating GH levels, and plasma IGF-1 concentrations, indicating that temperature-dependent endocrine regulation plays a central role in growth modulation [52]. Within a permissive thermal range, increased water temperature is generally accompanied by elevated IGF-1 expression and enhanced growth rates, as demonstrated in species such as rainbow trout [53]. However, this relationship is not linear, as exposure to suboptimal or stressful thermal conditions may result in elevated GH levels without a corresponding increase in IGF-1, reflecting reduced GH sensitivity and constrained somatic growth. Such temperature-induced trade-offs between stress tolerance and growth performance have been widely reported, particularly under conditions of limited energy availability or nutritional restriction [52,54]. Consequently, long-term adaptation to low-temperature environments may favor regulatory mechanisms that optimize energy allocation and partially mitigate growth suppression under cold conditions, rather than simply maximizing growth rate. In this context, the long-term residence of the HLJ population in the low-temperature and highly seasonal environment of the Heilongjiang basin may have contributed to population-specific regulatory patterns in growth- and metabolism-related pathways, potentially influencing both environmental adaptability and growth-related breeding potential. However, this possibility was not addressed in the present study and warrants further analysis and discussion.

## 5. Conclusions

In conclusion, this study compared four Mandarin fish populations collected from different river basins. Genetic diversity and population structure were analyzed, and growth-related SNP loci were developed and further characterized for their population specificity. Population genetic structure demonstrated that the four populations are genetically isolated and possess moderate levels of polymorphism. A total of 26 growth-related SNP loci were identified through GWAS, corresponding to 158 potential functional genes. These genes were primarily enriched in pathways including metabolic pathways, the NOD-like receptor signaling pathway, and necroptosis. Through non-tag SNP identification, this study newly identified 118 potentially linked loci, from which 13 SNPs significantly associated with body weight were further selected and their superior genotypes determined. Importantly, the study also revealed, for the first time, that the HLJ population possesses six population-unique high-growth genotypes, suggesting a substantial potential for growth-related selective breeding. However, these loci should be considered preliminary candidates rather than confirmed targets for selective breeding. Further studies involving multiple cohorts and within-population analyses are needed to determine whether the observed associations truly reflect genetic effects rather than population- or sampling-specific influences. Assessing the magnitude of these effects and their predictive value will provide more reliable guidance for their potential use in selective breeding. These findings provide essential data for evaluating geographical variation and facilitating growth-trait selection in Mandarin fish.

## Figures and Tables

**Table 1:** Significant SNP loci identified for body weight traits.

SNP	CHR	POS	REF	ALT	Method
Chr2_26654033_A_T	2	26654033	A	T	MLM
Chr2_26654919_A_C	2	26654919	A	C	MLM
Chr2_27438480_A_C	2	27438480	A	C	MLM
Chr3_2761657_T_C	3	2761657	T	C	FarmCPU
Chr4_12232732_T_G	4	12232732	T	G	MLM
Chr5_11085808_G_A	5	11085808	G	A	MLM
Chr8_3880709_T_C	8	3880709	T	C	FarmCPU
Chr9_27523974_C_G	9	27523974	C	G	MLM
Chr9_7377675_T_C	9	7377675	T	C	MLM
Chr10_27276393_A_G	10	27276393	A	G	MLM
Chr10_3129797_A_T	10	3129797	A	T	BLINK, FarmCPU, MLM
Chr11_14239856_T_A	11	14239856	T	A	MLM
Chr11_17764223_A_G	11	17764223	A	G	MLM
Chr11_22966715_G_C	11	22966715	G	C	MLM
Chr11_22971482_G_A	11	22971482	G	A	MLM
Chr13_21261594_G_A	13	21261594	G	A	MLM
Chr13_6999213_T_A	13	6999213	T	A	MLM
Chr14_23603173_T_C	14	23603173	T	C	MLM
Chr19_24824698_A_G	19	24824698	A	G	MLM
Chr20_18903685_T_C	20	18903685	T	C	MLM
Chr20_21148254_A_G	20	21148254	A	G	BLINK, MLM
Chr20_542292_A_G	20	542292	A	G	BLINK
Chr21_21195384_G_C	21	21195384	G	C	MLM
Chr22_6261294_A_G	22	6261294	A	G	MLM
Chr23_4132299_T_C	23	4132299	T	C	MLM
Chr23_4858864_A_G	23	4858864	A	G	FarmCPU

## Data Availability

The data presented in this study are openly available. Raw sequence reads are deposited in the SRA (accession number PRJNA1252799).

## Supplementary Materials

The following supporting information can be downloaded at: https://www.mdpi.com/article/10.3390/vetsci13010055/s1, Table S1. Summary of sequencing statistics for the 90 samples from four populations; Table S2. List of 158 potential functional genes identified within a ±50 kb window around each significant SNP; Table S3. Summary of 17,764 SNP loci detected within the 158 potential functional genes; Table S4. SNP loci potentially linked to the significant GWAS SNPs based on pairwise linkage disequilibrium analysis.
